# The Impact of Statins before High-Risk CABG on Postoperative Multiple Organ Function

**DOI:** 10.1155/2020/9519736

**Published:** 2020-01-14

**Authors:** Jiayang Wang, Wen Yuan, Kui Zhang, Nan Liu, Dong Liu, Yujie Zhou

**Affiliations:** ^1^Department of Cardiac Surgery, Beijing An Zhen Hospital Capital Medical University, Beijing 100029, China; ^2^Center for Cardiac Intensive Care, Beijing An Zhen Hospital Capital Medical University, Beijing 100029, China; ^3^Department of Cardiology, 12th Ward, Beijing Anzhen Hospital, Beijing Institute of Heart Lung and Blood Vessel Disease, Beijing Key Laboratory of Precision Medicine of Coronary Atherosclerotic Disease, Clinical Center for Coronary Heart Disease, Capital Medical University, Beijing 100029, China

## Abstract

**Background:**

The purpose of this cohort study was to investigate the independent relationship between preoperative statin therapy (PST) and postoperative severe multiorgan failure, measured by the Sequential Organ Failure Assessment (SOFA) maximum greater than 11, in high-risk patients undergoing isolated coronary artery bypass grafting (CABG).

**Methods:**

The present study is a perspective, single-center, cohort analysis enrolling high-risk patients undergoing CABG from Jan 1, 2018, to Dec 31, 2018, in Beijing Anzhen hospital.

**Results:**

Among a total of 880 high-risk patients undergoing isolated CABG included in this study, 503 (57.2%) experienced statin therapy before CABG. The SOFA maximum was significantly lower in the PST group compared with the control group (7.8 ± 3.0 v 9.2 ± 3.4, *P* < 0.0001). Multivariate logistic regression analysis demonstrated the incidence of the severe multiorgan dysfunction, measured by SOFA maximum ≥11, was dramatically reduced in the PST group (OR, 0.68, 95% CI 0.50–0.92, *P*=0.013). Furthermore, preoperative statin therapy (PST) might be associated with a decreased risk of postoperative major adverse cardiovascular and cerebral events and acute kidney injury, but an increased risk of postoperative hepatic inadequacy.

**Conclusion:**

SOFA maximum was significantly lower in the PST group compared with the control group and the incidence of the severe multiorgan dysfunction was dramatically reduced in the PST group. The findings of this study might shed new light on questions of positive or negative effects of PST on multiple organ function after high-risk CABG, so as to ultimately improve high-risk patient in-hospital outcomes from CABG.

## 1. Background

Previous studies demonstrated that the incidence of death in coronary artery bypass grafting (CABG) ranges from 2.94 to 32.5 according to different surgical severity and population [1, 2]. Therefore, it is essential to develop prognostic models for accurately identify mortality and morbidity after isolated CABG, especially high-risk CABG. Research data proved that the Sequential Organ Failure Assessment (SOFA) score per se as an independent risk factor for mortality after CABG and SOFA could be regarded as the most effective prognostic model for guiding the use of preventive and early therapeutic strategies to reduce mortality and morbidity for patients undergoing high-risk CABG [2, 3]. Recently, SOFA maximum is recommended to assess multiorgan dysfunction over time and severe multiorgan failure, measured by SOFA maximum greater than 11, predicted an in-hospital mortality of 95% [3, 4].

Preoperative statin therapy (PST) is known to be the most effective medications for cardiac surgical patients with hyperlipidemia [5]. However, the beneficial or detrimental effects of PST on cardiovascular and cerebral vascular, renal, respiratory and liver function in patients undergoing isolated high-risk CABG are still unclear. Results from studies that investigated the effects of PST on postoperative organ dysfunction are also controversial [6–9]. More importantly, previous studies only focused on one single organ, and no studies demonstrated the relationship between PST and severe multiple organ dysfunction after high-risk CABG. This may be ascribed to the lack of effective and comprehensive prediction models to evaluate postoperative multiple organ dysfunction. The appearance of SOFA maximum can solve this problem.

To fill the above knowledge gap, we systematically assessed the multiorgan function in high-risk patients undergoing isolated CABG in Beijing Anzhen hospital. The purpose of this cohort study was to investigate the independent relationship between PST and postoperative severe multiorgan failure, measured by SOFA maximum greater than 11, in high-risk patients undergoing isolated CABG and also examine the direct correlation between PST and the incidence of cardiac and cerebral vascular, respiratory, liver as well as renal postoperative complications. Recognition of the association and determinants of PST on postoperative multiple organ dysfunction should lead to strategies to improve the prognosis of patients undergoing elective high-risk CABG.

## 2. Methods

### 2.1. Study Population and Clinical Data

The present study is a perspective, single-center, cohort analysis enrolling high-risk patients undergoing CABG from Jan 1, 2018, to Dec 31, 2018, in Beijing Anzhen hospital. High-risk was defined as CHD patients with euroscore II of 6% or more. Patients received 20 mg of atorvastatin per day before CABG was included in the PST group. Patients undergoing CABG combined with other open-chest surgeries such as valvular repair or replacement were excluded. The gathered data included the main baseline clinical, echocardiographic, and procedural characteristics. Two reviewers (D.L. and W.Y.) independently extracted the above information. Informed consent was obtained from each patient on the day of admission. The ethical review and informed consent of this study were approved by the institutional ethics committee of Beijing Anzhen Hospital, Capital Medical University.

### 2.2. Definitions and Study Endpoints

Endpoints were: (1) in-hospital adverse outcomes defined as in the Society of Thoracic Surgeons (STS) national database [10]. The specific definitions are located on the STS website (http://www.sts.org/registries-research-center/sts-national-database/adult-cardiac-surgery-database/data-collection). A composite endpoint of in-hospital major adverse cardiovascular and cerebral events (MACCE) and the STS-defined variables of major morbidity were utilized; (2) in-hospital SOFA maximum: sequential assessment of in-hospital organ dysfunction is a good indicator of prognosis and SOFA, which is assessed in all patients after CABG every day, can help assess organ dysfunction or failure over time and are useful to evaluate morbidity [11]. The highest in-hospital SOFA score, namely SOFA maximum, of greater than 11 predicted a mortality of 95% [11, 12].

### 2.3. Statistical Analysis

Statistical analysis was performed using the SPSS version 25.0 statistical software (IBM Corporation, Armonk, New York, USA). Baseline characteristics were compared between the patients with major bleeding and without major bleeding. Continuous variables were expressed as mean value ± standard deviation and compared by the Student's *t*-test if normally distributed and otherwise as median (minimum, maximum) and compared by the Wilcoxon rank sum test. Categorical variables are expressed as percentages and were compared by the *χ*2 statistic or continuity-correction *χ*2 when cell counts were <5 or Fisher's exact test when cell counts were <1.

We used multivariable logistic regression analysis to investigate the association between PST and the incidence of SOFA maximum greater than 11, in-hospital postoperative MACCE, acute kidney injury (AKI), hepatic inadequacy, and infection adjusting for potential confounding factors. Forward stepwise selection was used to identify significant confounding variables. Potential confounders that had been reported in previous studies as important determinants of perioperative outcomes would be offered to the logistic regression models including: age, female gender, body mass index (BMI), diabetes, hypertension, prior MI, prior transient ischemic attacks (TIA) or cerebral vascular accident (CVA), current smoker, hypercholesterolaemia, previous peripheral vascular diseases (PVD), previous atrial fibrillation (AF), previous chronic obstructive pulmonary diseases (COPD), ventricular aneurysm, emergency CABG, decreased left ventricular ejection fraction (LVEF), euroscore II, duration of operation, off Pump CABG, New York Heart Association (NYHA) Functional Classification, and drugs before CABG. And closely associated factors (*P* < 0.05) from the univariate analysis were also included in the multivariable logistic regression analysis (*P* < 0.05 was retention criterion for each factor). Power of the association between risk factors and outcomes was expressed as odds ratio (OR).

All *P* values are 2-sided. Results were considered to be statistically significant at a *P* < 0.05.

## 3. Results

### 3.1. Demographic and Perioperative Characteristics

Among a total of 880 high-risk patients undergoing isolated CABG included in this study, 503 (57.2%) experienced statin therapy before CABG. The mean time of PST was 5.2 months. Baseline, procedural, and discharge data for the patients are shown in Table 1. Compared with the control group, significantly higher proportions of patients with PST had presented with the following clinical characteristics at hospital admission: male sex (69.2% v 46.9%, *P* < 0.0001), moderate and poor LVEF (48.1% v 40.8%, *P*=0.028), lower euroscore II (8.7 ± 4.4 v 8.8 ± 2.9, *P*=0.033), hypertension (59.8% v 43.0%, *P* < 0.0001), diabetes (56.1% v 23.6%, *P* < 0.0001), previous MI (16.9% v7.0%, *P* < 0.0001), preoperative angiotensin-converting enzyme inhibitors (ACEI)/angiotensin receptor blockers (ARB) (22.3% v 14.1%, *P*=0.002), and preoperative beta-blocker therapy (58.6% v 41.4%, *P* < 0.0001). On the contrary, the proportion of patients undergoing CABG without cardiopulmonary bypass was significantly lower in the PST group. Other characteristics were comparable in the two groups.

### 3.2. In-Hospital Outcomes

In-hospital outcomes for the two groups are shown in Table 2. The SOFA maximum was significantly lower in the PST group compared with the control group (7.8 ± 3.0 v 9.2 ± 3.4, *P* < 0.0001, Table 2). In addition, the proportion of patients with SOFA maximum greater than 11 was also significantly lower in the PST group. With respect to other secondary clinical outcomes, the rate of in-hospital MACCE, especially nonfatal stroke, acute kidney injury (AKI), and noninvasive ventilator, was significantly lower in patients with PST than in controls (Table 2). On the contrary, the rate of hepatic inadequacy postinfection was higher in the PST group (Table 2).

### 3.3. Multivariate Logistic Regression Analysis on Severe Multiorgan Dysfunction (Primary Endpoint)

Multivariate logistic regression analysis on the total patients demonstrated the incidence of the severe multiorgan dysfunction, measured by SOFA maximum ≥11, was dramatically reduced in the PST group (OR, 0.68, 95% CI 0.50–0.92, *P*=0.013, Table 3). On the contrary, female gender (OR, 1.93, 95% CI 1.43–2.60, *P* < 0.0001), higher euroscore II (OR,1.05, 95% CI 1.01–1.09, *P*=0.012), hypertension (OR, 1.40, 95% CI 1.02–1.87, *P*=0.021), previous MI (OR, 1.99, 95% CI 1.30–3.04, *P*=0.002), NYHA class III and IV (OR, 1.58, 95% CI 1.17–2.13, *P*=0.003), moderate and poor LVEF (OR, 2.38, 95% CI 1.76–3.21, *P* < 0.0001), emergency CABG (OR, 5.64, 95% CI 3.02–10.56, *P* < 0.0001), off Pump CABG (OR, 1.36, 95% CI (1.46–1.92), *P*=0.044), and longer duration of surgery (OR, 1.68, 95% CI 1.46–1.92, *P* < 0.0001) (Table 3) were the independent risk factors for severe multiorgan dysfunction.

### 3.4. Multivariate Logistic Regression Analysis on In-Hospital MACCE

Multivariate logistic regression analysis on the total patients demonstrated PST (OR, 0.60, 95% CI 0.44–0.81, *P*=0.001) may be associated with a decreased risk of in-hospital MACCE (Table 4). Besides, hypertension, higher euroscore II, current smoker, previous MI, previous TIA or CVA, previous AF, previous COPD, moderate and poor LVEF, NYHA III and IV, and longer duration of surgery were the independent risk factors for in-hospital MACCE (Table 4).

### 3.5. Multivariate Logistic Regression Analysis on AKI

Multivariate logistic regression analysis on the total patients demonstrated that PST (OR, 0.25, 95% CI 0.12–0.54, *P* < 0.0001) and ACE inhibitor or ARB may be associated with a decreased risk of postoperative AKI (Table 5). Besides, current smoker, emergency CABG, and longer duration of surgery were the independent risk factors for postoperative AKI (Table 5).

### 3.6. Multivariate Logistic Regression Analysis on In-Hospital Hepatic Inadequacy

Multivariate logistic regression analysis on the total patients revealed that the independent risk factors for in-hospital hepatic inadequacy were PST (OR, 1.49, 95% CI 1.01–2.18, *P*=0.042), hypertension, previous MI, previous PVD, and previous COPD. Besides, female gender and off Pump CABG may be associated with a decreased risk of in-hospital hepatic inadequacy (Table 6).

### 3.7. Independent Risk Factors for Postoperative Infection

Multivariate logistic regression analysis on the total patients revealed that the independent risk factors for postoperative infection were PST (OR, 2.09, 95% CI 1.42–3.08, *P* < 0.0001), BMI, DM, previous MI, a history of ventricular aneurysm, moderate and poor LVEF, NYHA III and IV, and emergency CABG (Table 7).

## 4. Discussion

This is the first study to prospectively explore the independent association between PST and postoperative severe multiorgan failure, measured by SOFA maximum greater than 11, in high-risk patients undergoing isolated CABG. Besides, we also examined the direct correlation between PST and the incidence of cardiac and cerebral vascular, respiratory, liver as well as renal postoperative complications. Our key findings are: (1) the SOFA maximum was significantly lower in the PST group compared with the control group and multivariate logistic regression analysis on the total patients demonstrated the incidence of the severe multiorgan dysfunction, measured by SOFA maximum ≥11, was dramatically reduced in the PST group; (2) PST might be associated with a decreased risk of postoperative MACCE and AKI, but an increased risk of postoperative hepatic inadequacy. Respiratory complications, such as hypoxemia, reintubation, and tracheotomy, were comparable in the PST and control groups; (3) PST was also independently associated with postinfection.

Although statin is used in a large proportion of patients before surgery, its potential impact on postoperative multiorgan function is still incompletely understood. With respect to renal function, Singh et al. found that PST reduced the CRRT and cardiac mortality significantly but exerted no effects on the incidence of AKI after CABG. [6] Wang et al. also demonstrated that PST may not reduce the risk of AKI in patients following isolated CABG. [13] On the contrary, Layton found that statin therapy immediately before CABG may modestly reduce the incidence of postoperative AKI, particularly in younger CABG patients. [14] Our previous evidence-based study including 59,771 patients also confirmed that PST significantly reduced the risk for postoperative AKI regardless of the types of diagnosis and staging criteria in cardiac surgical patients. [7] In addition, the cardiovascular protective effects of PST have already been well recognized. Knatterud et al. proved that PST delayed the progression of atherosclerosis and further reduced the risk for postoperative cardiovascular events in coronary heart disease patients following revascularization. [15] Furthermore, the uncertain safety of statin on liver function in high-risk CABG remains a major concern, and studies on statin-induced hepatotoxicity after high-risk CABG are sparse. A review preliminarily revealed the hepatotoxicity of statins and other lipid-lowering drugs. [16] They demonstrated that both simvastatin and atorvastatin have been correlated with more than 50 case reports of liver dysfunction and other statins have been implicated in this type of liver dysfunction as well. Another research found the association between dose escalation of atorvastatin and acute liver failure. [17] However, the adverse effects of PST on liver outcomes among cardiac surgical populations still need to be investigated further. Besides, respiratory complications after CABG are common, with an occurrence of 10 to 25% [18]. However, the independent relationship between PST and respiratory complications has not been confirmed because of a paucity of data [8]. Relevant high-quality prospective studies are still essential. Last but not least, neurologic complications, especially stroke, are associated with increased mortality and longer hospitalization [19]. However, previous research found that PST was not associated with a decreased risk for stroke and encephalopathy after high-risk CABG [20, 21]. The current research demonstrated that PST might be associated with a decreased risk of postoperative MACCE and AKI, but an increased risk of postoperative hepatic inadequacy. Respiratory complications, such as hypoxemia, reintubation, and tracheotomy, were comparable in the PST and control groups. More importantly, the incidence of the severe multiorgan dysfunction, measured by SOFA maximum ≥11, was dramatically reduced in the PST group compared with the controls.

Based on the above knowledge, PST has exerted a positive effect on cardiac, neurological, and renal function, and a negative effect on liver function after high-risk CABG. However, the association between PST and postoperative severe multiple organ dysfunction is still unknown. The current study first demonstrated that SOFA maximum was significantly lower in the PST group compared with the control group and the incidence of the severe multiorgan dysfunction, measured by SOFA maximum ≥11, was dramatically reduced in the PST group. The benefits of PST for cardiac dysfunction and mortality after high-risk CABG have been well established [8]. Recently, researchers have demonstrated that PST may benefit not only cardiac but also renal, neurological, and respiratory function. The pathophysiological mechanisms underlying the positive effects of PST on neurological, respiratory, and renal function might be closely related to the non-lipid-lowering activities of statins [22]. First of all, inflammation during CABG is reported to be a potential cause of organ dysfunction [23]. Previous studies proved PST could increase the release of anti-inflammation cytokines and reduce the levels of proinflammatory mediators, such as interleukin-6, interleukin-8, and tumor necrosis factor-*α* [9, 14]. In addition, ischemia-reperfusion injury as well as endothelial dysfunction are reported to be both independent associated with an increased risk of multiple organ dysfunction in patients undergoing high-risk CABG, especially on-pump surgery [24]. The pleiotropic effects of PST also include improvement in endothelial function and attenuation of reperfusion injury, which can decrease the risk of multiple organ dysfunction directly after CABG and further improve the prognosis of surgery [25, 26]. Recently, a high-quality prospective report confirmed that preoperative high-dose atorvastatin therapy could protect myocardium in patients following coronary revascularization by decreasing the risk of ischemia-reperfusion injury and endothelial damage during surgery [27]. Besides, the beneficial impacts of PST on multiple organ dysfunction might also be attributed to the following activity of statins: antithrombosis [7]. The above positive effects of PST on multiple organ function outweigh its side effects on liver function, leading to the incidence of the severe multiorgan dysfunction, measured by SOFA maximum ≥11, which was dramatically reduced in the PST group.

It is worth mentioning that the current study found that respiratory complications, such as hypoxemia, reintubation, and tracheotomy, were comparable in the PST and control groups. This might be due to the beneficial impacts of PST on respiratory dysfunction that cannot offset other etiologies-induced respiratory dysfunction [28]. Specifically, all of the prolonged mechanical ventilation, hypoxemia, reintubation, and tracheotomy might be regarded as a clinical endpoint of multiconditions including those that have no connection with the beneficial impacts of PST (lipid-lowering activities, anti-inflammation, antithrombosis, and improvement in endothelial function.), such as a prolonged residual anesthesia effect, a major or life-threatening bleeding as well as atelectasis. Those etiologies could offset the organ protective impacts of PST and may make the benefit of PST on respiratory function less noticeable than on a cardiac, neurological, and renal function [29, 30].

This study has several limitations. Firstly, the present study is a perspective, single-center, cohort analysis. High-quality, large-scale, and multicenter randomized controlled trials are required to further confirm the conclusion. Secondly, although SOFA maximum is recommended to assess multiorgan dysfunction over time and severe multiorgan failure, measured by SOFA maximum greater than 11, predicted an in-hospital mortality of 95%, we still need other organ failure assessments to measure severe multiorgan failure in order to verify each other. Thirdly, we confirmed that PST increase was associated with an increased risk of other endpoints, such as noninvasive ventilator. However, the independence of the correlation still needs further verification. Last but not least, the impact of PST on the long-term multiple organ function requires further examination.

## 5. Conclusions

This current observational cohort analysis demonstrated that PST might be associated with a decreased risk of postoperative MACCE and AKI, but an increased risk of postoperative hepatic inadequacy. Respiratory complications were comparable in the PST and control groups. In addition, SOFA maximum was significantly lower in the PST group compared with the control group, and multivariate logistic regression analysis on the total patients demonstrated that the incidence of the severe multiorgan dysfunction, measured by SOFA maximum ≥11, was dramatically reduced in the PST group. The findings of this study might shed new light on questions of positive or negative effects of PST on multiple organ function after high-risk CABG, and can ultimately improve high-risk patient in-hospital outcomes from CABG.

## Figures and Tables

**Table 1:** Characteristics of study population.

Variable	Preoperative statin therapy		*P*-value
	YES (*n* = 503)	NO (*n* = 377)	
Demographics			
Age, mean (SD), y	65.4 (7.6)	64.8 (10.9)	0.349
Male sex, *n* (%)	348 (69.2)	177 (46.9)	<0.0001
BMI, mean (SD), kg/m2	25.1 (2.9)	24.7 (3.4)	0.061
BMI≥30, *n* (%)	24 (4.8)	25 (6.6)	0.239

Medical history			
Hypertension, *n* (%)	301 (59.8)	162 (43.0)	<0.0001
HLP, *n* (%)	250 (49.7)	112 (29.7)	<0.0001
Diabetes mellitus, *n* (%)	282 (56.1)	89 (23.6)	<0.0001
Smoker, *n* (%)	149 (29.6)	91 (24.1)	0.079
COPD, *n* (%)	51 (10.1)	35 (9.3)	0.731
PVD, *n* (%)	116 (23.1)	72 (19.1)	0.116
Previous MI, *n* (%)	85 (16.9)	26 (7.0)	<0.0001
Previous CVA, *n* (%)	36 (7.2)	18 (4.8)	0.158
Previous AF, *n* (%)	68 (13.6)	62 (16.4)	0.250
LVEF, mean (SD), %	50.6 (15.1)	53.3 (19.1)	0.018
Moderate and poor LVEF (<50%) *n* (%)	242 (48.1)	154 (40.8)	0.028
Ventricular aneurysm, *n* (%)	53 (10.5)	25 (6.6)	0.055

Status			
Urgent CABG, *n* (%)	21 (4.2)	26 (6.9)	0.053
Euroscore II	8.7 (4.4)	8.8 (2.9)	0.033
NYHA class, *n* (%)			
II	310 (61.6)	255 (67.6)	0.076
III and IV	193 (38.4)	122 (32.4)	0.076
Off-pump	246 (48.9)	222 (58.9)	0.003
Duration of operation mean (SD), *h*	4.1 (6.4)	4.7 (1.4)	<0.0001

Medication at discharge			
ACEI/ARB	112 (22.3)	53 (14.1)	0.002
CCB	64 (12.7)	40 (10.6)	0.345
Aspirin	385 (76.5)	274 (72.7)	0.209
Beta-blocker	295 (58.6)	156 (41.4)	<0.0001

CABG: coronary artery bypass grafting; LVEF: left ventricular ejection fraction; BMI: body mass index; MI: myocardial infarction TIA: prior transient ischemic attacks; CVA: cerebral vascular accident; PVD: previous peripheral vascular diseases AF: previous atrial fibrillation; COPD: previous chronic obstructive pulmonary diseases; NYHA : New York Heart Association; ACEI: angiotensin-converting enzyme inhibitors; ARB: angiotensin receptor blockers; CCB: calcium channel blockers.

**Table 2:** In-hospital outcomes.

	PST (*n* = 503)	No PST (*n* = 377)	*P* value
SOFA Maximum ≥11	24.3% (122/503)	34.2% (129/377)	*P*=0.002
SOFA maximum	7.8 (3.0)	9.2 (3.4)	*P* < 0.0001
MACCE	27.6% (139/503)	35.0% (132/377)	*P*=0.022
In-hospital mortality	1.2% (6/503)	1.1% (4/377)	*P*=1.000
Nonfatal MI	4.6% (23/503)	4.8% (18/377)	*P*=1.000
Nonfatal stroke	0.6% (3/503)	2.9% (11/377)	*P*=0.011
New-onset AF	18.1% (91/503)	22.8% (86/377)	*P*=0.090
New-onset VA	6.6% (33/503)	11.4% (43/377)	*P*=0.015
Perioperative IABP	13.3% (67/503)	11.9% (45/377)	*P*=0.610
Perioperative ECMO	0.6% (3/503)	0.0% (0/377)	*P*=0.265
Reoperation	3.6% (18/503)	1.9% (7/377)	*P*=0.153
After infection	17.3% (87/503)	9.0% (34/377)	*P* < 0.0001
Pulmonary infection	16.1% (81/503)	9.0% (34/377)	*P*=0.002
After bloodstream infection	1.2% (6/503)	0.0% (0/377)	*P*=0.040
AKI	5.4% (27/503)	2.7% (10/377)	*P* < 0.0001
CRRT	2.8% (14/503)	1.3% (5/377)	*P*=0.165
Hepatic inadequacy	17.7% (89/503)	12.5% (47/377)	*P*=0.047
Hypoxemia	8.5% (43/503)	6.1% (23/377)	*P*=0.197
Noninvasive ventilator	2.0% (10/503)	8.0% (30/377)	*P* < 0.0001
Reintubation	2.2% (11/503)	2.4% (9/377)	*P*=1.000
Tracheotomy	1.0% (5/503)	0.0% (0/377)	*P*=0.075
ICU stay (Day)	2.7 (3.7)	2.4 (1.8)	*P*=0.130
Postoperative hospital stay (Day)	8.1 (5.4)	8.0 (3.0)	*P*=0.839
Cost (RMB)	138,636.1 (62,142.1)	137,345.6 (36,636.8)	*P*=0.720

MACCE: major adverse cardiovascular and cerebral events; MI: myocardial infarction; AF: previous atrial fibrillation; AKI: acute kidney injury; CRRT: continuous renal replacement therapy; IABP: intra-aortic balloon pump; ECMO: extracorporeal membrane oxygenation; SOFA : Sequential Organ Failure Assessment; VA: ventricular arrhythmias; PST: preoperative statin therapy.

**Table 3:** Independent risk factors for SOFA maximum greater than 11.

	Maximum SOFA score >11		*X* ^2^/*t*	*P* value	Multivariate analysis OR (95% CI)	*P* value
	YES (*n* = 251)	NO (*n* = 629)				
PST	122 (48.6)	381 (60.6)	10.5	0.013	0.68 (0.50–0.92)	0.013
ACE inhibitor or ARB	56 (22.3)	109 (17.3)	2.9	0.104		
Advanced age	66.0 ± 7.7	64.8 ± 9.6	1.8	0.070		
Female gender	130 (51.8)	225 (35.8)	19.1	<0.0001	1.93 (1.43–2.60)	<0.0001
Euroscore II	9.5 ± 3.8	8.5 ± 3.8	3.5	<0.0001	1.05 (1.01–1.09)	0.012
BMI	24.8 ± 3.2	25.0 ± 3.1	0.63	0.531		
Hypertension	149 (59.4)	314 (49.9)	6.4	0.014	1.40 (1.02–1.87)	0.021
Current smoker	61 (24.39)	179 (28.5)	1.6	0.241		
Previous MI	46 (18.3)	65 (10.3)	10.4	0.002	1.99 (1.30–3.04)	0.002
Previous TIA or CVA	9 (3.6)	45 (7.2)	3.9	0.061		
Hypercholesterolaemia	115 (45.8)	247 (39.3)	3.2	0.081		
Previous AF	39 (15.5)	91 (10.3)	0.16	0.675		
Previous COPD	17 (6.8)	69 (11.0)	3.6	0.060		
Ventricular aneurysm	20 (8.0)	51 (8.1)	0.35	0.601		
Moderate and poor LVEF (<50%)	152 (60.6)	244 (38.8)	33.5	<0.0001	2.38 (1.76–3.21)	<0.0001
NYHA III and IV	109 (43.4)	206 (32.8)	8.90	0.003	1.58 (1.17–2.13)	0.003
Emergency CABG	31 (12.4)	16 (2.5)	34.1	<0.0001	5.64 (3.02–10.56)	<0.0001
Off pump	147 (58.6)	321 (51.0)	4.1	0.044	1.36 (1.01–1.82)	0.044
Duration of operation (hours)	4.8 ± 1.5	4.2 ± 0.8	157.2	<0.0001	1.68 (1.46–1.92)	<0.0001

PST: preoperative statin therapy; CABG: coronary artery bypass grafting; LVEF: left ventricular ejection fraction; BMI: body mass index; MI: myocardial infarction; TIA: prior transient ischemic attacks; CVA: cerebral vascular accident; PVD: previous peripheral vascular diseases; DM: diabetes mellitus; NYHA : New York Heart Association; ACEI: angiotensin-converting enzyme inhibitors; ARB: angiotensin receptor blockers.

**Table 4:** Independent risk factors for in-hospital MACCE.

	In-hospital MACCE		*X* ^2^/*t*	*P* value	Multivariate analysis OR (95% CI)	*P* value
	YES (*n* = 271)	NO (*n* = 609)				
PST	139 (51.3)	364 (59.8)	5.5	0.022	0.60 (0.44–0.81)	0.001
ACE inhibitor or ARB	48 (17.7)	117 (19.2)	0.3	0.641		
Female gender	117 (43.2)	238 (39.1)	1.3	0.265		
Euroscore II	9.2 ± 5.0	8.6 ± 3.2	26.7	<0.0001	1.04 (1.01–1.08)	0.018
BMI	25.5 ± 3.8	24.7 ± 3.7	1.7	0.193		
Hypertension	178 (65.7)	285 (46.8)	26.8	<0.0001	2.41 (1.78–3.28)	<0.0001
DM	111 (41.0)	260 (42.7)	0.2	0.658		
Current smoker	96 (34.7)	144 (23.6)	13.1	<0.0001	1.60 (1.16–2.21)	0.004
Previous MI	45 (16.6)	66 (10.8)	5.7	0.021	1.57 (1.03–2.40)	0.035
Previous TIA or CVA	29 (10.7)	25 (4.1)	14.2	<0.0001	2.81 (1.60–4.93)	<0.0001
Previous PVD	67 (24.7)	124 (20.4)	2.1	0.157		
Hypercholesterolaemia	110 (40.1)	252 (41.2)	0.05	0.882		
Previous AF	55 (20.3)	75 (12.3)	9.5	0.003	1.67 (1.12–2.45)	0.011
Previous COPD	38 (14.0)	48 (7.9)	8.0	0.001	2.28 (1.42–3.66)	0.001
Ventricular aneurysm	19 (7.0)	59 (9.7)	1.7	0.247		
Moderate and poor LVEF (<50%)	146 (53.9)	250 (41.1)	12.4	0.001	1.79 (1.33–2.42)	<0.0001
NYHA III and IV	115 (42.4)	200 (32.8)	7.5	0.008	1.57 (1.17–2.11)	0.003
Emergency CABG	18 (6.6)	29 (4.8)	1.3	0.258		
Off pump	137 (56.5)	331 (49.8)	1.1	0.306		
Duration of operation (hours)	4.5 ± 1.3	4.3 ± 1.0	1.1	0.003	1.16 (1.02–1.32)	0.022

PST: preoperative statin therapy; CABG: coronary artery bypass grafting; MACCE: major adverse cardiovascular and cerebral events; LVEF: left ventricular ejection fraction; BMI: body mass index; MI: myocardial infarction; TIA: prior transient ischemic attacks; CVA: cerebral vascular accident; PVD: previous peripheral vascular diseases; DM: diabetes mellitus; NYHA : New York Heart Association, ACEI: angiotensin-converting enzyme inhibitors; ARB: angiotensin receptor blockers.

**Table 5:** Independent risk factors for AKI.

	AKI		*X* ^2^/*t*	*P* value	Multivariate analysis OR (95% CI)	*P* value
	YES (*n* = 37)	NO (*n* = 843)				
PST	10 (27.0)	493 (58.5)	14.3	<0.0001	0.25 (0.12–0.54)	<0.0001
ACE inhibitor or ARB	1 (2.7)	164 (19.5)	6.5	0.008	0.14 (0.02–1.01)	0.052
Female gender	17 (45.9)	338 (40.1)	0.5	0.497		
Euroscore II	7.8 ± 1.2	8.8 ± 3.9	17.7	0.112		
BMI	25.5 ± 3.3	24.9 ± 3.1	0.05	0.247		
Hypertension	24 (64.9)	439 (52.1)	2.3	0.134		
Current smoker	25 (67.6)	215 (25.5)	31.6	<0.0001	6.98 (3.40–14.34)	<0.0001
Previous MI	5 (13.5)	106 (12.6)	0.03	0.801		
Previous TIA or CVA	0 (0.0)	54 (6.4)	2.5	0.161		
Previous PVD	8 (21.6)	183 (21.7)	0.01	1.000		
Previous AF	7 (20.3)	123 (12.3)	0.5	0.476		
Previous COPD	5 (13.5)	81 (9.6)	0.6	0.397		
Ventricular aneurysm	19 (7.0)	59 (9.7)	1.7	0.247		
Moderate and poor LVEF (<50%)	15 (53.9)	381 (41.1)	0.3	0.615		
Emergency CABG	6 (16.2)	41 (4.9)	9.0	0.011	3.79 (1.50–9.58)	0.005
Off pump	24 (64.9)	444 (52.7)	2.1	0.178		
Duration of operation (hours)	6.9 ± 1.7	4.2 ± 0.9	44.5	<0.0001	4.47 (3.27–6.12)	<0.0001

PST: preoperative statin therapy; CABG: coronary artery bypass grafting; LVEF: left ventricular ejection fraction; BMI: body mass index; MI: myocardial infarction; TIA: prior transient ischemic attacks; CVA: cerebral vascular accident; PVD: previous peripheral vascular diseases; DM: diabetes mellitus; NYHA : New York Heart Association; ACEI: angiotensin-converting enzyme inhibitors; ARB: angiotensin receptor blockers.

**Table 6:** Independent risk factors for In-hospital hepatic inadequacy.

	In-hospital hepatic inadequacy		*X* ^2^/*t*	*P* value	Multivariate analysis OR (95% CI)	*P* value
	YES (*n* = 136)	NO (*n* = 739)				
PST	89 (65.4)	414 (56.3)	4.2	0.047	1.49 (1.01–2.18)	0.042
Female gender	35 (25.7)	315 (42.6)	13.7	<0.0001	0.47 (0.31–0.72)	<0.0001
Euroscore II	9.4 ± 5.4	9.0 ± 3.5	2.0	0.050		
BMI	24.2 ± 2.9	25.0 ± 3.1	−2.5	0.357		
Hypertension	93 (68.4)	365 (49.4)	16.6	<0.0001	2.18 (1.48–3.23)	<0.0001
Current smoker	40 (29.4)	200 (27.1)	0.3	0.601		
Previous MI	25 (18.4)	86 (11.6)	4.7	0.035	1.72 (1.05–2.80)	0.030
Previous TIA or CVA	8 (5.9)	46 (6.0)	0.02	1.000		
Previous PVD	40 (29.4)	151 (20.4)	5.4	0.024	1.63 (1.08–2.46)	0.020
Previous COPD	34 (25.0)	47 (6.9)	47.5	<0.0001	4.09 (2.47–6.78)	<0.0001
Ventricular aneurysm	7 (5.1)	71 (9.6)	2.8	0.103		
NYHA III and IV	51 (37.5)	264 (35.7)	0.2	0.698		
Emergency CABG	5 (3.7)	42 (5.7)	0.9	0.413		
Off pump	51 (37.5)	415 (56.2)	16.1	<0.0001	0.59 (0.40–0.87)	0.008
Duration of operation (hours)	4.5 ± 1.4	4.3 ± 1.0	1.8	0.079		

PST : Preoperative statin therapy; CABG: coronary artery bypass grafting; LVEF: left ventricular ejection fraction; BMI: body mass index; MI: myocardial infarction; TIA: prior transient ischemic attacks; CVA: cerebral vascular accident; PVD: previous peripheral vascular diseases; DM: diabetes mellitus COPD: previous chronic obstructive pulmonary diseases; NYHA : New York Heart Association.

**Table 7:** Independent risk factors for postoperative infection.

	Postoperative infection		*X* ^2^/*t*	*P* value	Multivariate analysis OR (95% CI)	*P* value
	YES (*n* = 121)	NO (*n* = 759)				
PST	87 (71.9)	416 (54.8)	12.5	< 0.0001	2.09 (1.42–3.08)	<0.0001
Female gender	42 (34.7)	313 (41.2)	1.8	0.195		
Euroscore II	8.4 ± 3.9	8.8 ± 3.8	−1.1	0.275		
BMI	25.5 ± 3.3	24.8 ± 3.1	2.3	0.024	1.07 (1.01–1.14)	0.024
Hypertension	68 (56.2)	395 (52.0)	0.7	0.433		
DM	70 (57.9)	301 (39.7)	14.2	<0.0001	1.76 (1.17–2.65)	0.007
Previous MI	26 (21.5)	85 (11.2)	10.0	0.003	1.73 (1.03–2.89)	0.038
Previous TIA or CVA	11 (9.1)	43 (5.7)	2.1	0.153		
Previous PVD	22 (18.2)	169 (22.3)	1.0	0.344		
Hypercholesterolaemia	43 (35.5)	319 (42.0)	1.8	0.196		
Previous AF	18 (14.9)	112 (14.8)	0.001	1.000		
Previous COPD	6 (5.0)	80 (10.5)	3.7	0.068		
Ventricular aneurysm	30 (24.8)	48 (6.3)	44.1	<0.0001	4.44 (2.65–7.43)	<0.0001
Moderate and poor LVEF (<50%)	72 (59.5)	324 (42.3)	11.6	0.001	1.82 (1.22–2.72)	0.003
NYHA III and IV	69 (57.0)	256 (33.7)	27.5	<0.0001	2.60 (1.75–3.86)	<0.0001
Emergency CABG	13 (10.7)	34 (44.8)	8.1	0.008	2.02 (1.01–4.04)	0.047
Off pump	63 (52.1)	405 (53.4)	0.07	0.845		
Duration of operation (hours)	4.2 ± 1.0	4.4 ± 1.1	−1.1	0.305		

PST: preoperative statin therapy; CABG: coronary artery bypass grafting; LVEF: left ventricular ejection fraction; BMI: body mass index; MI: myocardial infarction; TIA: prior transient ischemic attacks; CVA: cerebral vascular accident; PVD: previous peripheral vascular diseases; DM: diabetes mellitus; NYHA : New York Heart Association.

## Data Availability

No data were used to support this study.

## Abbreviations

PST: Preoperative statin therapy
CABG: Coronary artery bypass grafting
CHD: Coronary heart disease
MACCE: Major adverse cardiovascular and cerebral events
SOFA: Sequential organ failure assessment
LVEF: Left ventricular ejection fraction
BMI: Body mass index
MI: Myocardial infarction
TIA: Prior transient ischemic attacks
CVA: Cerebral vascular accident
PVD: Previous peripheral vascular diseases
AF: Previous atrial fibrillation
COPD: Previous chronic obstructive pulmonary diseases
NYHA: New York Heart Association
AKI: Acute kidney injury
CRRT: Continuous renal replacement therapy
IABP: Intra-aortic balloon pump
ECMO: Extracorporeal membrane oxygenation
ACEI: Angiotensin-converting enzyme inhibitors
ARB: Angiotensin receptor blockers.
